# Upsetting offsetting? Nathan the Wise’s Ring Parable and three reasons why not to adopt the carbon offsetting logic to biodiversity

**DOI:** 10.1007/s13280-024-01994-w

**Published:** 2024-02-25

**Authors:** Ludovico Giacomo Conti, Peter Seele

**Affiliations:** Università della Svizzera italiana, Via Buffi 13, 6900 Lugano, Switzerland

**Keywords:** Biodiversity, Biodiversity crisis, Carbon, Climate change, Offsetting, Ring Parable

## Abstract

The climate crisis and the alarming loss of biodiversity require urgent action. One of the most popular tools to tackle these crises is offsetting, an accounting logic through which environmental damages can be compensated elsewhere with environmental benefits. Developed to help address the climate crisis, the carbon offsetting logic has also been transposed to address biodiversity loss. Biodiversity offsets permit the compensation of residual environmental damages through equivalent gains undertaken elsewhere. This article identifies three arguments that show why such a transposition is problematic. To explain the fundamental problem with biodiversity offsetting, the famous Lessing’s “Nathan the Wise” Ring Parable (*Ringparabel*) is proposed as an allegoric interpretation of the biodiversity offsetting logic, stressing that unique entities lose their uniqueness and *power* once people try to replicate them.

## Offsetting as the panacea?

Dubai’s COP28 began shortly after the release of the UN *Broken Record* report, which underscored the inadequacy of countries’ commitments to address the global climate crisis. The report heightened expectations for COP28 to serve as a pivotal opportunity for correcting course and accelerating sustainability transformations. Nevertheless, COP28’s outcome fell short, achieving merely a “transitioning away from fossil fuels” (Morton et al. 2023)—a result well below the phase-out or phase-down demanded by civil society and scientists. Such an outcome will likely place even greater reliance on mechanisms such as carbon offsetting to remain within the boundaries set by the Paris Agreement—a trend endorsed by influential figures such as John Kerry (US climate envoy), Ursula von der Leyen (EU commission president) and Tony Blair (UK former prime minister), who, at the COP28, sought to rekindle discussions on the carbon credit market (Greenfield and Harvey 2023).

Although COP28’s outcome could be defined as a first step in the right direction, apprehensions arise regarding the escalating use of and dependence on offsetting. This overuse may convey the wrong message to those involved in other environmental debates—particularly those centred on biodiversity protection. It may insinuate that *partial* protection of biodiversity (akin to transitioning or phase-down strategies for fossil fuels) supplemented by offsetting is sufficient—thereby obviating the necessity for comprehensive protection (analogous to a phase-out from fossil fuels). This paper cautions against this overreliance on offsetting strategy and the transposition of the offsetting logic to the realm of biodiversity. The argument presented in this short communication challenges the idea that the quantitative logic of CO2 offsetting that applies in the world of physics and free markets can be transposed to, for example, a specific forest with possibly hundreds of species and millions of living organisms per square km.

The argument is outlined as follows. First, the basic functioning of carbon offsetting is explained, and its transposition to the realm of biodiversity is presented. Then, three reasons are offered to underscore the inherent flaws in this transposition. Acknowledging the intricacies of the debate, an allegoric interpretation based on Gotthold Ephraim Lessing’s “Nathan the Wise” (1779) Ring Parable (*Ringparabel*) is proposed to delineate the complexities and nuances surrounding the use of biodiversity offsets.

## From carbon offsetting to biodiversity offsetting

Carbon offsetting can be engaged in because of ethical willingness, marketing reasons, or to comply with legal constraints. It is an instrument that allows individuals, companies, or states to contribute to environmental schemes to balance out some or all of their emissions. This can be achieved by removing carbon dioxide from the atmosphere or reducing emissions (Hyams and Fawcett 2013). The offsetting process, both in its regulated and voluntary form, operates on an accounting logic where damages are balanced out by equivalent gains so as “to make no long-term net contribution to atmospheric greenhouse gas concentrations” (Hyams and Fawcett 2013, p. 91). This commensurability is made possible by the non-localised nature of climate change as “greenhouse gases spread evenly throughout the atmosphere, so reducing them anywhere contributes to overall climate protection” (Stockholm Environment Institute 2011). Trading of emissions occurs at various scales through certificate exchange markets, such as the Carbon Offsetting and Reduction Scheme for International Aviation (CORSIA), the European Emission Trading System (EU ETS) for European countries, or the Clean Development Mechanism (CDM) established in 1997 with the Kyoto Protocol (Lovell 2010). Nevertheless, its most known application to the general public might be the compensation for CO2 emissions from flying by purchasing a carbon credit, according to the distance and type of plane, linked to a carbon removal project (such as afforestation projects) that compensates the emissions produced (IATA 2022).

This accounting logic is being transposed to address the unprecedented ecosystem degradation caused by biodiversity loss (Ceballos et al. 2015). At the intergovernmental level, offsetting has been listed in Target 19(d) of the Kunming-Montreal Global Biodiversity Framework (GBF) as a tool and solution to “progressively increase the level of financial resources” for achieving the four framework’s long-term goals by 2030 (CBD 2022, p. 5). At the business level, these commitments are being translated into more comprehensive standards. For example, the Global Reporting Initiative (GRI)—a global standard for corporate sustainability reporting—is expanding its ESG reporting standards with a revised section that considers organisations’ impacts on biodiversity. In it, biodiversity offsets are identified as a means to compensate for residual negative impacts (GSSB 2022). Here, biodiversity offsetting has become a prominent market-based instrument for biodiversity conservation.

Biodiversity offsetting is an umbrella term that encompasses several compensatory, mitigatory and conservatory policies (Bull et al. 2013; Hrabanski 2015). It is carried out according to the principle of “No Net Loss” (NNL), which aims to balance the environmental impacts of, for example, infrastructural projects with “mitigation, restoration, or compensation efforts to ensure that no overall environmental loss results” (Morseletto 2022, p. 2167). Within this framework, residual environmental damages that cannot be avoided, minimised, or remediated, such as the destruction of habitat, killing of species, or removal of ecosystem functionalities (Spash 2015), are compensated through positive “real, additional, quantifiable, permanent, verifiable, and enforceable” (Ruseva 2023, p. 62) equivalent gains undertaken elsewhere (Morseletto 2022). These gains could be in the form of recovery, regeneration, security, or an increase in biodiversity (Bekessy et al. 2010). Hence, the biodiversity offsetting rationale transforms conservation efforts into “a technical accounting exercise by converting impacts and restoration/improvement into common units, i.e. creating full commensurability” (Spash 2015, p. 547) that are then sold and bought in trading systems such as, among others, the Wetland Banking in the USA and the biobanking in Australia (Hrabanski 2015). Additionally, biodiversity restoration and conservation can also be traded on “the secondary market for commercial debt” (Bedarff et al. 1989, p. 445) through debt for nature swaps. Presented in the 1980s by Thomas E. Lovejoy, nature swaps are a complex financial instrument used to refinance countries’ sovereign debt by selling part of this debt at a discount and then using the money saved from this deal to restore or protect the environment (Bedarff et al. 1989).

When done correctly, biodiversity offsetting could create, restore, or protect areas that would have been otherwise damaged or lost (concept of *averted loss*) (Devenish et al. 2022). Caution is warranted, however, to avoid that the scandals that hit the carbon offsetting market (Benedito and Sarmiento 2022) spill over to the biodiversity offsetting market. The media investigation undertaken by the Guardian, Die Zeit, and SourceMaterial, published in January 2023, exposed how Verra, the leading certifier for the voluntary market, generated so-called “phantom credits” (Greenfield 2023). The accusation is rooted in the process of averted loss: Verra emitted credits from rainforest areas, claiming that, based on current trends, those areas would have been cut down but for its conservation efforts. By securing them, Verra would have been entitled to emit credits equal to the amount of CO2 conserved in the forest (Greenfield 2023). However, the media investigation discovered that such threatened areas have never been under threat in the first place and, therefore, would not have been eligible for the creation of carbon credits (Padin-Dujon 2023).

## Three reasons not to adopt the carbon offsetting logic

Having explained the functioning of carbon and biodiversity offsetting, three arguments are presented to raise doubt about the transferability of the accounting logic from carbon offsetting to biodiversity.

First, ecosystems “differ in type, location, time, or ecological context” (Bull et al. 2013, p. 4). Therefore, to establish commensurability among diverse entities and locations, biodiversity offsetting relies on proxies (Humphries et al. 1995), also known as “surrogates” (Maron et al. 2016). Although such a strategy permits comparing apples to oranges, the practical impediment in devising comprehensive and applicable metrics introduces ambiguity in accounting practices, hinders the comparison of losses and gains, and complicates the achievement of no net loss (Bull et al. 2013).

The difficulty is grounded in the struggle to balance simplicity and complexity. The first is crucial for facilitating exchanges but prone to a “crude simplification of the natural world” (Maron et al. 2016)—thus potentially rendering metrics deficient in important aspects. The second enhances the inclusivity of different aspects but also risks of transforming them into “black boxes” (Maron et al. 2016). These difficulties are exacerbated by our limited understanding of the intricate relationships among ecosystem components, making it challenging to find comparable and sufficient offsets to compensate for biodiversity losses.

To counter this deficiency, some have advocated for multipliers, a factor that increases the gains by changing the 1:1 ratio in favour of a 1:x > 1 one. In economic terms, multipliers would be equivalent to applying “a discount rate to calculate net present value” (Bekessy et al. 2010, p. 153). A practical example of the use of multipliers is South Africa’s Western Cape offset scheme, according to which, for every hectare of “critically endangered ecosystems” lost, there must be a compensation of thirty hectares elsewhere (ratio 1:30) (DEADP, 2007, p. iv). Nevertheless, it has been shown that these ratio “corrections” are difficult to calculate precisely—oftentimes they are calculated on “rules of thumb” (Moilanen et al. 2009, p. 470)—and exact values might even be impossible to compute (Laitila et al. 2014).

Second, ecosystems are complex systems that evolved over millions of years in which many specific variables and entities interact (climate, soil, nutrients, species, individuals, and symbiosis). This ontological complexity entails that not only it is problematic to compensate for the loss of one ecosystem with gains in another, but also is compensating for losses caused to the ecosystem *X* by restoring it to the state *ex ante* the damage. Contrary to expectation, restoration does not imply reinstating previous functionalities (Ambrose 2000).

The notion that the ecosystem *X* will never precisely revert to its original state is underscored by the intrinsic difficulties in replicating the criteria and richness of the original natural ecosystem within biodiversity copies—since, “unlike a building that can be retrofitted for sustainability, once habitat is destroyed, it might be impossible to reconstruct” (Bekessy et al. 2010, p. 152). This is because two places will never have the same ecological and biodiversity features (Maron et al. 2016). As such, producing an exact replica cannot be physically or financially achieved (Wilkins et al. 2003).

Even in a hypothetical scenario where exact replication could be accomplished, ethical and practical issues would remain. The temporal lags inherent in offset schemes, delineated by Bull et al. (2013), introduce significant intergenerational ethical concerns. Moreover, on a practical level, these temporal discrepancies create inefficiencies, establishing a flawed mechanism wherein losses are certain, while gains are prospective and delayed into the future (Maron et al. 2016).

Third, if biodiversity offsetting is understood as a mere accounting computation, it can easily become an ethical bailout justified by a perverse financial calculus. The ability to pay for offsets thus becomes a license to continue harming nature (Karlsson and Edvardsson Björnberg 2021). This would mistakenly imply that by paying, one absolves one’s environmental damages and, as such, does no harm and no injustice (Broome 2012).

## Nathan the Wise’s ring parable as an interpretation

Although the idea of addressing climate change and biodiversity loss through one and the same instrument is appealing, this article argues that offsetting should not be used to address biodiversity losses. This is because the logic of carbon offsetting, akin to an accounting exercise (harm = compensation) made possible by the *non-localised* nature of climate change, is unsuitable for addressing the *geo-localised* issue of the biodiversity loss crisis.

To better explain why such a transposition should not be further encouraged, the famous “Ring Parable” (*Ringparabel*) of Gotthold Ephraim Lessing’s “Nathan the Wise” (1779) is proposed as an allegoric interpretation. In Lessing’s parable, a father has a ring with the power to make his owner pleasing in the eyes of God. Unsure about which of his three sons should inherit the ring, he calls a goldsmith and makes him cast two identical copies of the original ring so that each son would receive a ring. The sons, all believing to have the real ring, start to quarrel and resolve to ask a judge to set the case and decide which one has the real ring. The wise judge admonishes the three heirs for fighting over the real ring and states that the power of the original ring must have gone lost since all three sons were involved in the dispute.

The same occurs with biodiversity offsetting. Once biodiversity (here representing the original ring with its “power”, in our example the holistic uniqueness of biodiversity at a given place) is replicated through offsets and multipliers (akin to creating the rings in the parable), its “power” is lost. In fact, biodiversity offsetting causes a loss in biodiversity that cannot be recreated since with “biodiversity loss, there is no return to the *status quo ante*” (Ekins and Zenghelis 2021, p. 953), much like the inability to restore the power of the original ring in the parable.

Like Lessing’s parable, this article is a cautionary tale: relying on biodiversity offsetting to balance out future residual damages would equate to replicating the rings, a strategy that will decrease biodiversity—though “sold” as saving it.
